# Ten years of life in compost: temporal and spatial variation of North German *Caenorhabditis elegans* populations

**DOI:** 10.1002/ece3.1605

**Published:** 2015-07-15

**Authors:** Carola Petersen, Manja Saebelfeld, Camilo Barbosa, Barbara Pees, Ruben Joseph Hermann, Rebecca Schalkowski, Eike Andreas Strathmann, Philipp Dirksen, Hinrich Schulenburg

**Affiliations:** Department of Evolutionary Ecology and Genetics, Zoological Institute Christian-Albrechts UniversityAm Botanischen Garten 1-9, 24118, Kiel, Germany

**Keywords:** *Bacillus thuringiensis*, *Caenorhabditis elegans*, microsatellites, natural variation, population genetics, *Serratia*

## Abstract

The nematode *Caenorhabditis elegans* is a central laboratory model system in almost all biological disciplines, yet its natural life history and population biology are largely unexplored. Such information is essential for in-depth understanding of the nematode's biology because its natural ecology provides the context, in which its traits and the underlying molecular mechanisms evolved. We characterized natural phenotypic and genetic variation among North German *C. elegans* isolates. We used the unique opportunity to compare samples collected 10 years apart from the same compost heap and additionally included recent samples for this and a second site, collected across a 1.5-year period. Our analysis revealed significant population genetic differentiation between locations, across the 10-year time period, but for only one location a trend across the shorter time frame. Significant variation was similarly found for phenotypic traits of likely importance in nature, such as choice behavior and population growth in the presence of pathogens or naturally associated bacteria. Phenotypic variation was significantly influenced by *C. elegans* genotype, time of isolation, and sampling site. The here studied *C. elegans* isolates may provide a valuable, genetically variable resource for future dissection of naturally relevant gene functions.

## Introduction

The nematode *Caenorhabditis elegans* is one of the most intensively studied laboratory model systems, yet we still lack functional information on a large proportion of its genes (Petersen et al. 2015). One likely reason is that the nematode is almost exclusively studied under artificial laboratory conditions using a single natural isolate, the Bristol strain N2, which itself shows comprehensive adaptations to the laboratory environment (McGrath et al. 2009, 2011; Weber et al. 2010; Andersen et al. 2014). Genetic variants encountered in natural *C. elegans* populations and the naturally relevant traits are usually not considered during the comprehensive analyses on *C. elegans* gene functions. At the same time, however, we only have very little information of the nematode's natural ecology and population biology, which could be used as reference points for such analyses. Most of our current understanding of *C. elegans* ecology stems from work in recent years and was mainly focused on European and to some extent North American populations.

These recent studies highlighted that *C. elegans* is common in ephemeral habitats, especially in decaying plant material such as rotting fruits and stems from particular plants that are all rich in microbes (Barrière and Félix 2005a, 2007; Félix and Duveau 2012; Petersen et al. 2014). Anthropogenic habitats such as compost heaps similarly seem to represent a favorable environment, because they can harbor dynamic yet often stable *C. elegans* populations (Barrière and Félix 2007; Félix and Braendle 2010; Félix and Duveau 2012; Petersen et al. 2014, 2015). *Caenorhabditis elegans* has been found all over the world (Hodgkin and Doniach 1997; Barrière and Félix 2005b; Haber et al. 2005). The overall worldwide genetic diversity seems to be comparatively low, while local genetic diversity levels can reach similar values (Sivasundar and Hey 2003; Barrière and Félix 2005a, 2007; Haber et al. 2005; Cutter 2006; Rockman and Kruglyak 2009; Andersen et al. 2012), suggesting high mutation rates and, more likely, high immigration rates at single locations (Barrière and Félix 2005a), possibly in combination with a recent worldwide selective sweep (Andersen et al. 2012). The presence of *C. elegans* seems to be influenced in some populations by humidity and temperature (Félix and Duveau 2012; Petersen et al. 2014). Furthermore, the nematode expresses distinct behavioral responses toward naturally co-existing microbes (Schulenburg and Müller 2004; Volkers et al. 2013). Natural genetic variation is also found in resistance toward different pathogens such as *Bacillus thuringiensis* (Schulenburg and Müller 2004; Volkers et al. 2013) or viruses (Ashe et al. 2013), suggesting that these may exert high selective pressure in nature. Similar variation among natural isolates has also been identified for several other life-history traits, for example, different environmentally dependent influences on fecundity (Harvey and Viney 2007; Diaz and Viney 2014), generation and developmental time (Volkers et al. 2013), copulatory plug formation (Hodgkin and Doniach 1997; Rockman and Kruglyak 2009), dauer formation (Green et al. 2013), or male frequency and mating ability (Hodgkin and Doniach 1997; Teotónio et al. 2006; Wegewitz et al. 2008; Anderson et al. 2010), indicating that these may similarly be subject to natural selection. To date, however, the number of populations with sufficiently large sample size, repeated sampling time points, collected substrate types, and considered environmental parameters is still comparatively small, limiting the generality of the current findings.

The aim of this study is thus to enhance our understanding of *C. elegans* natural ecology and population biology by assessing genetic and phenotypic variation across time and space for two intensively sampled North German populations. *Caenorhabditis elegans* isolates were taken from our collections at two compost heaps in either Kiel or Roxel (Haber et al. 2005; Petersen et al. 2014). For one of these, Roxel, we were in the unique position to assess variation among samples collected 10 years apart from each other (2002 vs. 2011/2012), covering a minimum number of approximately 200 generations, assuming 4 months per year suitable for reproduction at an average temperature of 15°C (thus approximately 20 generations per year). For both sites, we additionally examined short-term changes, using samples from three time points within a 1.5-year period, covering approximately at least 40 generations (as two summers and autumns were included). We analyzed microsatellites to explore population genetic differentiation across time and space. We additionally focussed on two phenotypic traits, which are of likely relevance under natural conditions: population growth and bacterial choice behavior. Population growth represents an informative proxy for fitness, especially for a pioneering species in ephemeral habitats such as *C. elegans*. Choice behavior is likely of key importance in natural environments, in which differentiation between harmful and beneficial microbes is essential for nematode survival. These traits were evaluated in the presence of the standard laboratory food *Escherichia coli* and also several naturally associated bacteria.

## Materials and Methods

### Nematode and bacterial strains

The considered 137 natural strains of *C. elegans* used for the genotypic characterization (Table S1) were isolated from compost at two North German locations in 2002 and additionally between July 2011 and December 2012, as previously described (Haber et al. 2005; Petersen et al. 2014). The two sampling locations are the botanical garden in Kiel (54°20′N and 10°06′E; only the recent time points; Table S1) and a private garden in Roxel (51°57′N and 7°32′E; all time points; Table S1), which are found in a distance of about 300 km from each other. Eighty-five of the obtained strains were completely independent as they were isolated from separate substrate samples (Table S1). For the phenotypic characterization, 49 of these strains (33 completely independent) were analyzed for their population growth rate and 59 (37 completely independent) were analyzed for their choice behavior (Table S1). The standard *C. elegans* laboratory strains N2 and CB4856 were additionally included and were originally obtained from the CGC (Caenorhabditis Genetics Center), which is funded by NIH (National Institutes of Health) Office of Research Infrastructure Programs (P40 OD010440). Before the start of the experiments, all strains were thawed from frozen stocks, bleached, and grown on NGM (nematode growth medium), following standard procedures (Stiernagle 2006).

For the phenotypic assays, five different bacteria were used. The two Gram-negative bacteria *Serratia* sp. and *Serpens* sp. are natural isolates and co-occurred with *C. elegans* in compost samples from Kiel and Roxel, respectively. Each of the two bacteria was isolated from particular compost substrate samples, which also contained *C. elegans* in the two locations. The bacteria were cultured for 2 days at 25°C on LB agar, and fresh colonies were used to produce liquid cultures in LB medium at 37°C overnight. The *E. coli* strain OP50 was used as control for these two bacteria and cultured in LB medium at 37°C overnight. Furthermore, two strains of the Gram-positive bacterium *B. thuringiensis* were used: The nematocidal strain MYBT18247 (in the following: BT247) originally provided by the Agricultural Research Service Patent Culture Collection (United States Department of Agriculture, Peoria, IL) and the non-nematocidal strain DSM 350 (in the following: DSM350), obtained from the German Collection of Microorganisms and Cell Cultures (Deutsche Sammlung von Mikroorganismen und Zellkulturen GmbH, DSMZ, Braunschweig, Germany). Spore–toxin mixtures of BT247 and spore-only cultures of DSM350 were prepared before the start of the experiment and frozen in aliquots at −20°C, as described and established previously (Leyns et al. 1995; Hasshoff et al. 2007; Schulte et al. 2010). BT247 was used at a concentration of 1.9 × 10^9^ particles/mL and DSM350 at 6.3 × 10^8^ particles/mL.

### Microsatellite analysis

DNA of 139 *C. elegans* strains (137 natural isolates, N2, CB4856) was isolated using a modified CTAB- (Cetyl Trimethyl Ammonium Bromide-) based protocol (Schulenburg et al. 2001; Haber et al. 2005). A total of 400 *μ*L CTAB buffer and 2 *μ*L proteinase K (20 mg/mL; Thermo Scientific, Waltham, MA, USA) were added to a worm pellet originating from one plate full of starving L1 larvae and digested overnight at 50°C. Subsequently, 4 *μ*L RNase A (100 mg/mL; Qiagen, Hilden, Germany) was added, the samples were vortexed and incubated for 5 min at room temperature. DNA was extracted with 2 volumes of chloroform: isoamylalcohol (24:1), followed by centrifugation for 5 min at 17949 g. The top phase was mixed with 2/3 volumes of ice-cold 100% isopropanol and incubated at −20°C for 1 h, followed by centrifugation for 30 min at 17949 g, and subsequent washing of the DNA pellet in 1 mL 70% ethanol. The DNA pellet was air-dried and resuspended in 100 *μ*L TE buffer.

*Caenorhabditis elegans* genotypes were analyzed using six microsatellites, one each on chromosomes II, III, V, and X (*II-R, 3003, V-L* and *X-R)* and two on chromosome IV (*4001* and *IV-L*) (Schulte et al. 2010). Microsatellites were amplified in 20 *μ*L volumes containing 2 *μ*L 10× DreamTaq buffer (Thermo Scientific), 0.4 *μ*L dNTPs (10 mmol/L), 0.8 *μ*L of each primer (10 *μ*mol/L), whereby one primer per pair was fluorescently labeled, 0.1 *μ*L DreamTaq polymerase, 1 *μ*L template DNA (20 ng DNA/*μ*L), and 14.9 *μ*L distilled water. The primer sequences were published previously (Schulte et al. 2010). The cycling profile consisted of an initial denaturation for 2 min at 95°C, 35 cycles of 30 sec at 95°C, 30 sec at 60°C, and 30 sec at 72°C, followed by 45 min at 72°C final extension. Fragment size was assessed on an ABI PRISM 3730xl Genetic Analyzer (Applied Biosystems, Waltham, MA), using Peak Scanner Software v1.0 (Thermo Scientific, Waltham, MA, USA).

### Phenotypic analysis

#### Population growth rate

The population growth of 49 *C. elegans* natural isolates and the wild-type strain N2 was measured as produced offspring per initial worm, using 6-cm PFM (peptone-free medium) plates with a 400 *μ*L bacterial lawn (OD 7) of either *E. coli*, *Serratia* sp., or *Serpens* sp., or alternatively, a 500 *μ*L bacterial lawn of either DSM350 or BT247. In the latter experiments with *B. thuringiensis,* both strains were always mixed with OP50 (OD 5) in a ratio of 1:200 to provide sufficient amounts of food, which is especially required in case of the pathogenic strain. For each replicate, three hermaphrodites at the fourth larval stage (L4) were picked onto the bacterial lawns. In all experiments, we ensured that only hermaphrodites but not males were transferred in order to avoid any biases. Moreover, the populations were always initiated with three transferred hermaphrodites, in order to minimize the stochastic variations among replicates from the same treatment, which we previously observed to be much higher when populations were started with only a single worm. After 5 days at 20°C (approximately encompassing two offspring generations of the initial L4 hermaphrodites), the worms were washed off the plates using 1 mL M9 buffer including 0.1% Triton and directly frozen at −80°C until sample scoring. Dead worms were clearly distinguishable from living worms on pathogenic BT247 (Leyns et al., 1995 ), while this differentiation was not relevant for worms grown on nonpathogenic bacteria. The total number of offspring per strain was extrapolated from three counted replicates of 5–10 *μ*L and subsequently divided by the three initially used L4 hermaphrodites. The produced offspring per worm was compared for five replicates per worm isolate. The assay was performed without current knowledge of strain identity, and all treatment combinations were evaluated in parallel and in randomized order to avoid observer bias. The two possible food bacteria *Serratia* sp. and *Serpens* sp. were compared with *E. coli* OP50, whereas the pathogenic BT247 was compared with the non-nematocidal DSM350.

#### Choice behavior

The choice assay was performed with 49 natural *C. elegans* isolates and N2 and the bacteria *E. coli* OP50, *Serratia* sp., *Serpens* sp., BT247, and DSM350. Twenty-five microliters of *Serratia* sp. or *Serpens* sp. (OD 1) was pipetted to one side of a 9-cm PFM plate, and OP50 (OD 1) was pipetted to the other side. Plates with two *E. coli* spots were used as a control. Twenty-five microliters of BT247 or DSM350 were pipetted to one side of a plate and DSM350 to the opposite side. For the *B. thuringiensis* treatments, plates with two DSM350 spots were used as a control.

Ten hermaphroditic L4 worms were picked to the center of each plate. Experiments were always initiated with ten rather than single worms, because in our experience, the joint analysis of worm groups reduces stochastic variation among replicates of the same treatment. The number of worms in contact with either bacterial spot was counted after 14 h and 24 h. A CI (choice index) was calculated as a ratio: CI = [no. of worms at test bacterium – no. of worms at control]/[total no. of nematodes at test bacterium and control]. The CI represents the proportion of worms that preferred a particular test bacterium over the control. It can vary between +1 and −1, where −1 indicates choice of the control bacterium (either OP50 or DSM350) and +1 indicates the choice of the tested bacterium (either *Serratia* sp., *Serpens* sp., or BT247). A CI of zero indicates that there was no preference. The experiment was performed at 20°C with five replicates per treatment and strain. The test was performed without current knowledge of the *C. elegans* and bacterial isolate to avoid observer bias. The two possible food bacteria *Serratia* sp. and *Serpens* sp. were compared with *E. coli* OP50, whereas the pathogenic BT247 was compared with the non-nematocidal DSM350.

### Statistics

We used microsatellite data for a general characterization of the population genetics and focused on three main objectives: (1) analysis of overall genetic differentiation among the included data subsets, (2) fine-scale analysis of genetic differentiation between locations and sampling time points, and (3) visualization of the genetic relationships using a genotype network. Statistical analysis of overall population genetic differentiation was performed using an AMOVA (analysis of molecular variance) as implemented in the Arlequin software package v3.5.1.3 (Excoffier et al. 1992, 2007) and one data set containing all considered subpopulations (see Table 1). For the more detailed analysis of differentiation across time and space, we calculated pairwise *F*_ST_ values, using Arlequin v.3.5.1.3. For both AMOVA and the pairwise *F*_ST_, statistical significant was inferred using permutation tests (1023 or 110 permutations, respectively) as implemented in Arlequin. A heat map of the pairwise *F*_ST_ values was produced with Arlequin and the R statistical platform (version 2.13.0). A minimum spanning tree was inferred from the pairwise *F*_ST_ with Arlequin and then further adjusted to show alternative connections between genotypes using the FigTree Software v1.4.0.zip and Microsoft Office PowerPoint (version 2003).

**Table 1:** Data subsets used for the overall population genetic analysis using analysis of molecular variance

Location	Groups	Subpopulation.	Time period^1^	Number of isolates
Roxel	1	R0	2002	19
	2	R1	Late 2011	20
		R2	Mid 2012	20
		R3	Late 2012	19
Kiel	3	K1	Late 2011	20
		K2	Mid 2012	20
		K3	Late 2012	19

1 See also Table S1.

All statistical analyses of phenotypic variation were performed using the R platform (version 3.0.3). Separate nested analyses of variance were used to infer the effect of sampling location (collections from Kiel and Roxel 2011/2012), sampling time (collections obtained in Roxel 2002 and 2011/12), and genotype on different phenotypic measurements. Each bacterial treatment was analyzed separately and corrected for multiple comparisons by adjusting the *P*-values with the FDR (false discovery rate; Benjamini and Hochberg 1995). To ensure data comparability across runs, results of the population growth assay were standardized by dividing the number of offspring per worm, of each natural isolate, by the mean number of offspring per worm of the control under the respective test conditions (N2 on OP50 or DSM350, respectively). For the choice assay, data were standardized by subtracting the median of the control (N2 on OP50 or DSM350, respectively) from the recorded median of each natural isolate under the respective test conditions. To minimize the influence of random effects, we only considered replicates where at least five worms could be scored. We tested for correlations between CI values and the mean relative population growth using Spearman's rank test. We corrected for multiple comparisons by adjusting the *P*-values with FDR.

## Results

### Genotypic variation

**Figure 1:**
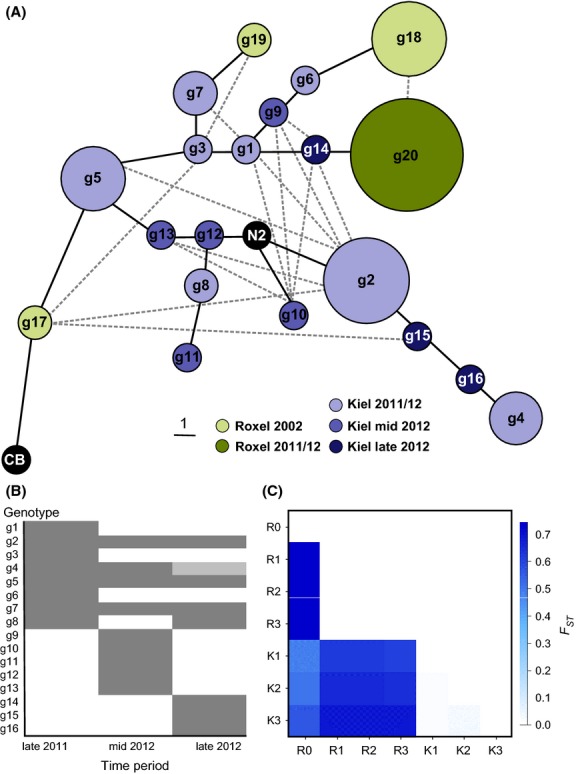
Microsatellite analysis of the North German populations across time and space. (A) Minimum spanning network of the genotype relationships. The strains N2 and CB4856 were included as a reference. The network was inferred using *F*_ST_ as a measure for genetic distance. Branch lengths correspond to the number of different alleles (see scale in legend) and circle sizes correspond to the number of isolates per genotype. Dashed lines indicate alternative connections between genotypes. The Kiel genotypes are given in different shades of purple depending on their occurrence across the time periods where light purple includes genotypes either found in only late 2011 and/or one or both of the later time periods; the genotypes that were unique in mid-2012 and late 2012 are given in purple and dark purple, respectively. Green color indicates genotypes from Roxel. (B) Occurrence of genotypes within Kiel across time. Genotype g4 in late 2012 appeared as one allele combination in the heterozygous genotype g15 and is thus given in light gray. (C) Genetic differentiation of subpopulations as *F*_ST_ values where 0 indicates the absence of differentiation (see right scale). The long-term comparison between Roxel 2002 and all subpopulations of Roxel 2011/12 and Kiel 2011/12 showed significant differences (all *P* < 0.001). All 2011/12 subpopulations from Kiel versus Roxel differed significantly from each other (all *P* < 0.001). No significant difference was found in the short-term comparisons among the subpopulations from Roxel 2011/12, whereas there was a trend for differences among the subpopulations from Kiel 2011/12 (all *P* < 0.1).

One hundred and thirty-nine *C. elegans* strains, including 137 natural isolates from either Kiel or Roxel in Germany, the Bristol strain N2, and the Hawaiian strain CB4856, were analyzed for differences in their genotypes at six microsatellite loci (Tables S1–S3). Among the natural isolates, a total of 20 genotypes were identified, all of which differed from the genotypes of the reference strains N2 and CB4856 (Fig. 1A). Four of the six microsatellites used in this study were analyzed before for the strains from Roxel isolated in 2002 and also for CB4856 (Haber et al. 2005). In general, the results of Haber et al. could be confirmed. A minor difference was observed in four natural isolates with a repeat number of eight instead of seven at locus IV-L. Moreover, for locus IV-L, we found 48 repeats instead of nine in strain CB4856, and 80 instead of seven in strains MY2 and MY23 (Haber et al. 2005). The new values were confirmed by repetition of the fragment analysis. The exact reasons for such variations are thus not really clear. They could be due to usage of different fragment analysis platforms, different polymerase chain reaction chemistry, and/or rapid mutation of the studied microsatellites. We decided to continue with the entire data set, because the majority of the 2002 strains did not differ between studies and because the observed differences did not lead to different genotype assignments. Nevertheless, we repeated the statistical analysis with and without the differences and obtained essentially identical results for population genetic differentiation. For simplicity, we only show the results for the entire data set.

All 59 isolates from Roxel from 2011/2012 had the same genotype g20, which was found for none of the other subpopulations (Table S1). Three genotypes (g17, g18, and g19) were found in the isolates from Roxel from 2002 with g18 being most frequent. The populations from Kiel showed 16 different genotypes (g1–g16). There were no shared genotypes among the Roxel populations from 2002 and 2011/2012, and no genotypes were shared between the populations from Kiel and Roxel. Only a single isolate, MY2530 from Kiel (genotype g15), showed heterozygosity, in this case, at three of six loci. This heterozygote could have resulted from a cross between g2 and g4, which jointly possess all of the alleles present in g15 (Table S2) and which are also adjacent to the heterozygote in the genotype network (Fig. 1A). Several genotypes within the Kiel population were shared between the time periods while others were unique within single time periods (Fig. 1B).

### Population genetic analysis

The Kiel population contained a higher level of diversity at both the number of genotypes (16 genotypes) and the calculated gene diversity (0.7752; computed according to Nei 1987) than the Roxel population (four genotypes; gene diversity of 0.3921 for the total of Roxel 2002 and 2011/2012; see summary in Table 2). The relationship of the identified genotypes is depicted in Figure1A.

**Table 2:** Characteristics of microsatellite variation for different data subsets

Subset	*N*^1^	Genotypes^2^	Alleles^3^	Loci^4^	*D* ± SD^5^	*H*_obs_^6^
R0	19	3	3	6	0.36 ± 0.09	0.0
R1–3^7^	59	1	1	0	0.0	0.0
Roxel overall	78	4	3	6	0.39 ± 0.04	0.0
K1	20	8	3.5	5	0.81 ± 0.04	0.0
K2	20	9	3.5	5	0.79 ± 0.04	0.0
K3	19	7	3	5	0.70 ± 0.07	0.05
Kiel overall	59	16	4	5	0.78 ± 0.03	0.02
Roxel + Kiel	137	20	5	6	0.76 ± 0.02	<0.01

1 Number of isolates within subset (see Table S1).

2 Number of genotypes.

3 Median number of alleles over all loci.

4 Number of polymorphic loci.

5 Gene diversity (=expected heterozygosity) ± standard deviation according to Nei (1987).

6 Observed heterozygosity.

7 The subpopulations of Roxel 2011/2012 (i.e., R1–R3) are combined as they all contain the same single genotype.

We here performed a focused analysis of population genetic differentiation among the locations, time periods, and subpopulations. AMOVA demonstrated significant genetic differentiation within and among the populations (1023 permutations; Fig. 1A; Table 3). Most of the variation was distributed among groups (63.37%, *P* = 0.0196) and among subpopulations (64.1%, *P* < 0.0001). The variation among subpopulations within groups was significant (1.99%, *P* = 0.0499). Pairwise comparison of *F*_ST_ values between the seven subpopulations and a permutation test (110 permutations) showed highly significant differences between the subpopulations from Roxel from 2002 and from 2011/12 (*P* < 0.0001; Fig. 1C). As the Roxel population was homogenous in 2011/12, there were no differences in the *F*_ST_ values between the subpopulations (*P* = 0.991). Within Kiel, the three time periods showed some variation, although the differences were not significant (0.1 > *P* > 0.05). All combinations of subpopulations among Kiel and Roxel differed significantly from each other (*P* < 0.0001).

**Table 3:** Analysis of molecular variance

Source of variation^1^	df^2^	*F*^3^	*P*^4^
Among groups	2	*F*_CT_ = 0.63	**0.0196**
Among subpopulations Within groups	4	*F*_SC_ = 0.02	**0.0499**
Among subpopulations	267	*F*_ST_ = 0.64	**<0.0001**
Total	273		

Values considered as significant at a significance level of 0.05 are given in bold.

1 Structure of data set: groups are defined by location and sampling time point: 1 = R0; 2 = R1, R2, R3; 3 = K1, K2, K3; subpopulations within groups refer to the three sampling time points R1, R2, R3 for Roxel and K1, K2, and K3 for Kiel (see Tables 1 and S1).

2 Degrees of freedom.

3 Fixation indices calculated over all loci as defined by (Weir and Cockerham 1984).

4 Probability of homogeneity between subsets calculated from 1023 permutations.

### Phenotypic variation in natural *C. elegans* populations

#### Population growth rate

**Figure 2:**
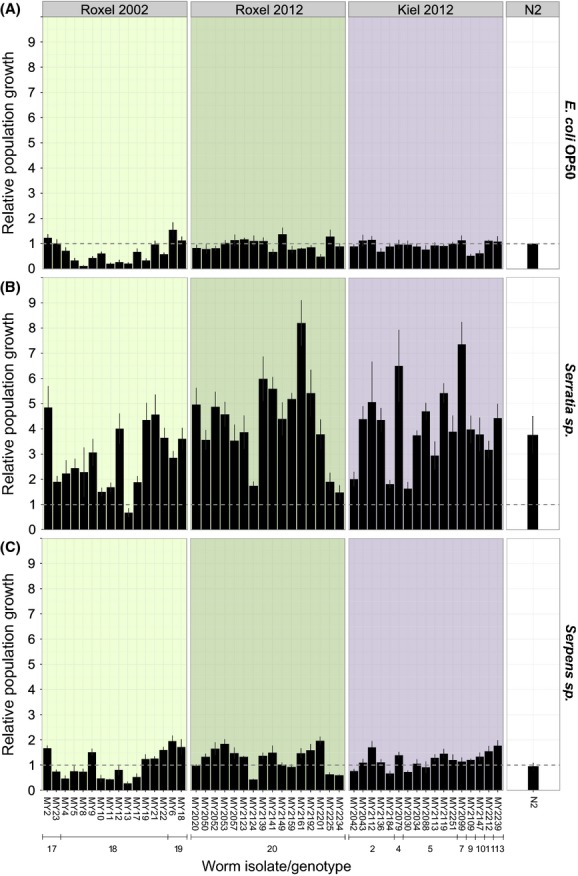
Population growth of natural *Caenorhabditis elegans* isolates. The population growth rate of the strains from Roxel (2002 and 2012) and Kiel (2012) and the wild-type strain N2 on (A) *Escherichia coli*, (B) *Serratia* sp.*,* and (C) *Serpens* sp. was analyzed after 5 days and is shown as mean population growth per initial worm relative to the mean population growth of N2 on OP50 per initial worm (indicated as dashed line). The error bars indicate standard error of the mean. Genotype numbers below strain designations refer to those from Table S2.

**Figure 3:**
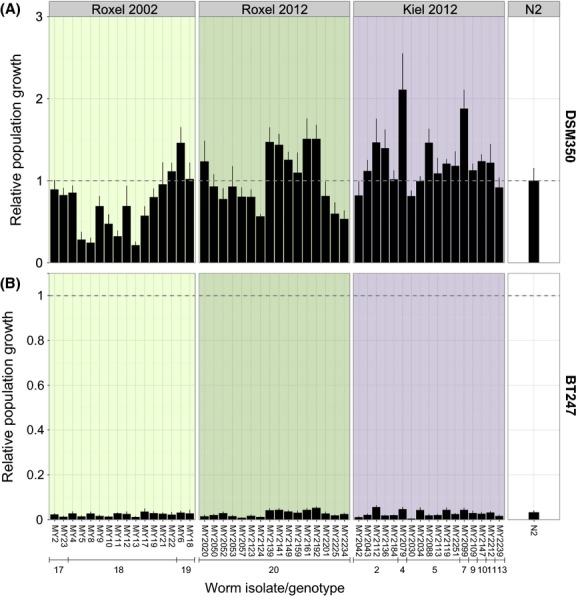
Population growth of natural *Caenorhabditis elegans* isolates. The population growth rate of the strains from Roxel (2002 and 2012) and Kiel (2012) and the wild-type strain N2 was analyzed on (A) *Bacillus thuringiensis* DSM350 and (B) BT247 after 5 days and is shown as mean population growth per initial worm relative to the mean population growth of N2 on DSM350 per initial worm (indicated as dashed line). The error bars indicate standard error of the mean. Note that, the scales of DSM350 and BT247 differ. Genotype numbers are given below strain codes and are identical to those in Table S2.

The population growth rate showed substantial variation among the 49 tested natural isolates of *C. elegans* and was influenced by several factors, including sampling site, genotype, and time of isolation (Figs. 2, 3; Table S4). On *Serratia* sp., *Serpens* sp., OP50, and DSM350, the differences could be explained by the worm isolate (all *P* < 0.001; see detailed results in Table S4), the sampling time (all *P* < 0.04), and the genotype (all *P* < 0.001). The factor sampling site was significant for DSM350 (*P* = 0.005) but for none of the other of the bacterial treatments. On BT247, worm isolate had a significant influence on the observed variation (*P* < 0.001), whereas the influence of the factor genotype was still indicated by a statistical trend (*P* = 0.083). On this bacterium, sampling time had no significant effect. Moreover, the natural isolates generally varied from the reference strain N2 (Table S5): 26.5% of the natural isolates differed significantly from N2 in their population growth rate on *E. coli* OP50 (all *P* < 0.037), 36.7% on *Serratia* sp. (all *P* < 0.042), 51.2% on *Serpens* sp. (all *P* < 0.049), 28.6% on DSM350 (all *P* < 0.048), and 36.7% on BT247 (all *P* < 0.045).

#### Choice behavior

**Figure 4:**
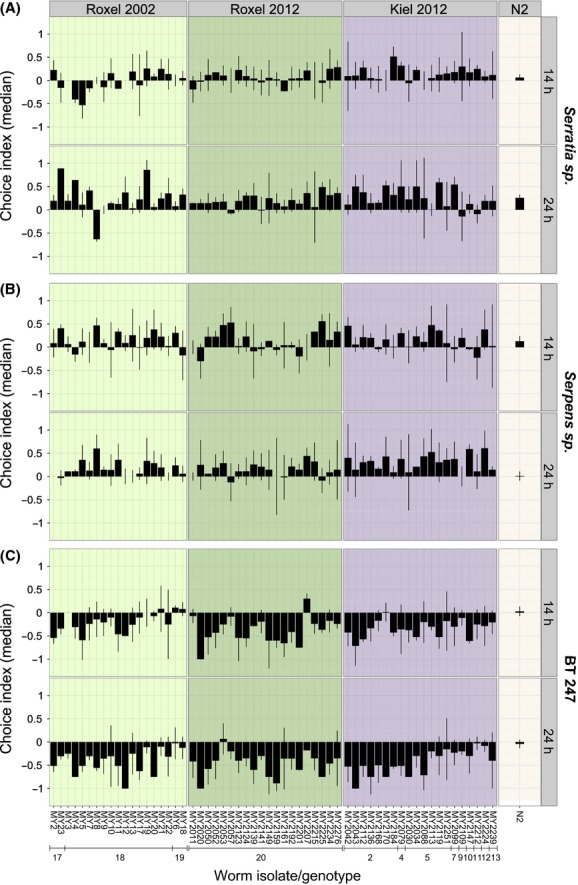
Choice behavior of natural *Caenorhabditis elegans* isolates. The choice behavior of strains from Roxel (2002 and 2012) and Kiel (2012) and the wild-type strain N2 was analyzed on *Serratia* sp. (A), *Serpens* sp.*,* (B) and BT247 (C) after 14 and 24 h. The bars show medians and the error bars median absolute deviation (mad). Genotype numbers are given below strain designations and are identical to those from Table S2.

The natural *C. elegans* strains showed significant differences in their attraction responses (Fig. 4; Table S6). Within the bacterial treatments, the factors worm isolate, sampling time, or genotype significantly affected variation in attraction, whereas the sampling site had no significant influence.

On BT247, the worm isolate significantly influenced the variation at time point 14 h (*P* = 0.012) and 24 h (*P* < 0.001), sampling time influenced attraction at 14 h (*P* = 0.014), and genotype produced a statistical trend at 14 h (*P* = 0.067) and was significant at 24 h (*P* = 0.001). On *Serpens* sp.*,* there was a trend for the factor genotype at time point 24 h (*P* = 0.073). Other factors showed no significant influence on choice behavior in the *Serpens* sp. treatment. There was no significant variation in the *Serratia* sp. treatment. In general, the preference for *Serratia* sp. and *Serpens* sp. increased over time while BT247 was more disliked over time (Fig. 4). In detail, most worm strains showed a neutral response and only some a preference toward both *Serratia* sp. and *Serpens* sp. after 14 h, whereas almost all strains preferred the two bacteria after 24 h. In contrast, the vast majority of strains disliked BT247 at both 14 and 24 h. The choice behavior differed between the natural isolates and N2 (Table S7). Seven percent or 10.2% of the natural strains differed significantly from N2 in their choice behavior on *Serratia* sp. after 14 h (*P* < 0.045) and 24 h (*P* < 0.039), respectively. On *Serpens* sp., 11.9% of the natural strains showed significant differences compared to N2 after both 14 h (*P* < 0.045) and 24 h (*P* < 0.045). 31.6% or 53.5% of the natural strains differed from N2 in the choice behavior toward BT247 after 14 h (*P* < 0.042) or 24 h (*P* < 0.041), respectively.

### Correlation between population growth rate and choice behavior

**Figure 5:**
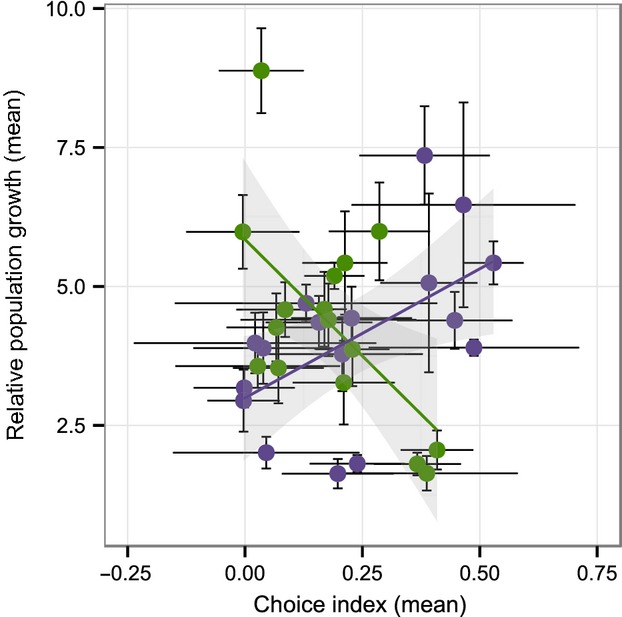
Correlation between population growth rate and choice index on *Serratia* sp. The natural *Caenorhabditis elegans* isolates were collected in 2012 in Kiel (purple dots) and Roxel (green dots). Lines are predicted from a linear model. Shaded areas indicate the 95% confidence interval. Error bars denote standard error of the mean.

We found a significant positive correlation between population growth rate and choice behavior on *Serratia* sp. for the isolates from Kiel isolated in 2012 (*P* < 0.026; Fig. 5; Table S8) and a negative trend in the corresponding treatment for the isolates from Roxel isolated in 2012 (*P* = 0.078). None of the other tested treatment combinations yielded a significant correlation.

## Discussion

We here provide a unique data set on spatial as well as long-term and short-term temporal variation in natural populations of the model nematode *C. elegans*. Our study demonstrates significant differentiation at both genetic and phenotypic levels, which we studied for life-history characteristics likely of high relevance in nature, such as population growth and bacterial choice behavior. Our findings highlight the presence of substantial variation in this model organism, which is usually unexplored in the large majority of studies with this nematode, as most studies focus on a single strain, N2. The data and especially the strain material presented here provide a valuable resource for future functional genetic analysis of environmentally relevant traits.

### Long-term, short-term, and spatial genetic differentiation

Although worm isolates from the same location were previously not available from two distant time points, one previous study still identified significant genetic differentiation across time when grouping isolates from different European locations that were either collected early versus those collected later (Haber et al. 2005). In this previous study, the significant time effect could thus also have been produced by some form of geographical variation. In our current study, there is no doubt that the 10-year time period significantly affected genetic composition at the tested isolation site in Roxel. These genotypic differences between 2002 and 2011/2012 could be a consequence of variation across time in environmental factors, chance fluctuations in the genotypic composition of the populations, and/or incomplete sampling. In the former two cases, the early genotypes may have gone extinct followed by recolonization of the compost by g20. Alternatively, the only genotype in the 2011/12 samples, g20, could have directly evolved from the most frequent genotype from 2002, g18, as indicated by a direct alternative connection in the genotype network (Fig. 1A). g20 may also have been overlooked in 2002 due to a then possible lower frequency and incomplete sampling of the population. In the subsequent 10 years, g20 would then have outcompeted the other genotypes or replaced these as a consequence of drift.

Next to long-term genetic differentiation, we also identified a trend for short-term variations in the samples from Kiel. Here, some genotypes did persist across the three sampling times, whereas several others were only present at restricted time periods. The pattern in Kiel clearly contrasted with the Roxel samples from the same short-term time period, where not a single change was observed. A similar pattern of populations with either significant short-term turnover of genotypes or the absence of temporal variation was previously reported for French locations, indicating a dynamic metapopulation structure of locations with extinctions followed by recolonization events and others with population stability (Barrière and Félix 2007). In the former case, new genotypes may arise through immigration, which is generally assumed to be mediated via nematode–invertebrate associations (Baird 1999; Caswell-Chen 2005; Félix and Braendle 2010). In our study locations in Kiel and Roxel, *C. elegans* was indeed found in association with slugs and isopods (unpublished data), these invertebrates could thus have acted as vectors. Immigration may also be mediated by vertebrate vectors and/or human activities (Andersen et al. 2012). Alternatively, it is also possible that some of the novel genotypes in Kiel are directly derived from previously present genotypes, as possibly indicated by small genetic distances among some of the genotypes (Fig. 1A).

Next to temporal variation, we also identified strong spatial genetic differentiation between the two sampled locations. This finding is again consistent with the previously studied French metapopulation (Barrière and Félix 2007) and a separate comparison between two French sites (Volkers et al. 2013), indicating that some geographic barrier and/or different population histories can create genetically distinct populations at different locations. The latter alternative is likely influential for the North German populations, because these were found to be subject to different population histories (see above). Moreover, *C. elegans* is generally believed to be able to spread wide distances with the help of vectors and/or human activities (Félix and Braendle 2010; Andersen et al. 2012). At the same time, it is nevertheless interesting to note that the two locations did not share even a single genotype. Thus, it is possible that there is some type of geographical barrier relevant for the nematodes between the two tested North German locations. Without doubt, we still require further long-term analyses of a larger number of locations at varying distances and a more extensive sampling of genotypes from individual sampling points, in order to fully understand the nematode's population biology.

### Phenotypic variation

Our phenotypic analysis focused on two traits of likely relevance under natural conditions. The population growth rate represents a composite measure of fitness, which combines reproductive rate, developmental time, and nematode survival and which is likely of high importance in ephemeral habitats, where high population growth rates are likely to determine the competitive success. Similarly, behavioral choice of suitable versus detrimental bacterial lawns is likely a key determinant of fitness in the wild, as it determines whether the worms have access to highly nutritious food organisms or are exposed to harmful pathogens. Both traits show substantial variation across the natural isolates and for the different tested bacteria. The results obtained in the presence of the pathogen were least variable for the particular nematode isolates and thus likely most informative. The factor *C. elegans* genotype had a significant and generally the strongest effect on the observed variation for the considered trait–bacteria combinations (Tables S4 and S6) – the main exceptions being population growth on nematocidal *B. thuringiensis* and choice behavior with *Serratia* sp. and *Serpens* sp. The next most influential factor was sampling time, which may at least partially be due to a difference in genotypes across time. These observations, especially the strong influence of genotype, demonstrate that genotypic differentiation translates into phenotypic variation. They additionally indicate that the considered traits or at least some related function may be under diversifying selection in nature, as expected for changing environmental parameters as often the case for pathogens. A similarly strong influence of genotype on natural variation was previously reported for related life-history traits using distinct sets of natural isolates, for example, population growth of French isolates on the same nematocidal *B. thuringiensis* strain BT247 (Volkers et al. 2013). Interestingly, in contrast to our results, choice preferences for natural food bacteria was previously found to be influenced by genotype for two French populations (Volkers et al. 2013), indicating that the selective consequences of food organisms may vary among populations but also among the food bacteria considered. Note that the previous study tested four very distinct bacteria, *Erwinia rhapontici*, *Sphingobacterium* sp., *Rhodococcus erythropolis*, and *Lactococcus lactis*, which were commonly found in the French locations (Volkers et al. 2013).

The bacterial community may generally be of key importance for *C. elegans*' life history and fitness in nature, as they include beneficial food microbes and possible pathogens (Petersen et al. 2015). As such, we may also expect different adaptations to alternative bacterial environments. This was indeed previously observed for the two compared French locations, which differed in their preference for the naturally co-occurring bacteria (Volkers et al. 2013). Interestingly, we found that the relationship between population growth rate on *Serratia* sp. and the choice of *Serratia* sp. showed a significant positive correlation for the isolates from Kiel, but a statistical trend for a negative correlation for the Roxel strains isolated in the same time period. The *Serratia* sp. bacterium was originally isolated from the same compost as the Kiel strains, which may thus have specifically adapted to this bacterium. The correlation between the two fitness-relevant traits, population growth rate, and behavioral choice may thus be a consequence of local adaptation to a common food source in Kiel (Fumagalli et al. 2011; Hancock et al. 2011). A similar correlation is not shown by the Roxel worms, which are likely exposed and possibly adapted to other bacteria not considered in the current study.

It is furthermore interesting to note that a large proportion of strains differed from the wild-type strain N2, especially in population growth on the various bacteria and also in the choice behavior toward the pathogenic BT247. The latter case is particularly surprising: Whereas the vast majority of natural isolates avoided BT247, N2 responded more or less neutrally. These observations may be due to comprehensive adaptation of N2 to the laboratory environment, which, for example, has led to comprehensive changes in genome sequence (Weber et al. 2010), in O_2_ chemosensation, associated aggregation behavior, and reproductive rate, which are most likely pleiotropically mediated by the N2 allele of the neuropeptide receptor gene *npr-1* (McGrath et al. 2009; Andersen et al. 2014). As the laboratory environment lacks pathogens and a diverse array of food bacteria, the N2 strain may have lost its ability to specifically respond to these bacteria, especially pathogens, and/or to efficiently use them as a source of nutrition. If it is true, then the genes involved in the interaction with naturally associated bacteria may not easily be inferred with N2.

In conclusion, a more detailed sampling of *C. elegans* only began within the past decade and for most parts of the world only a few natural isolates are as yet available (Barrière and Félix 2005a, 2007; Caswell-Chen 2005; Félix and Duveau 2012; Petersen et al. 2014). A larger number of independent isolates have so far only been obtained from French and German locations (Barrière and Félix 2005a, 2007; Haber et al. 2005; Félix and Duveau 2012; Petersen et al. 2014). We here provide a genetic and phenotypic examination of strain material from two of these locations and highlight the presence of comprehensive differentiation across time and space, affecting phenotypes of likely relevance under natural conditions. The characterized strains show clear differences to the canonical laboratory strain N2 and may thus be of high value for future dissection of genotype–phenotype interactions in natural populations and especially the gene functions of relevance in nature.

At the same time, our study also highlights the limitations of the current work on *C. elegans* ecology. For a more precise understanding of the temporal dynamics in *C. elegans* populations, we require a more continuous sampling across short and long time periods rather than sampling at only distinct time points as in the current study. For a detailed characterization of geographic variation, we similarly need a more comprehensive sampling across space, covering a continuum from microscale variation within a particular habitat to spatial differences within a defined geographic region (i.e., the North German plain which is generally similar in climate and habitat diversity) up to that across regions, countries, and continents. To date, we similarly do not know how complete and unbiased current sampling efforts are, especially in relation to the exact population size at a particular location. The main current constraint is the isolation procedure, which takes time and usually relies on an attractant such as the *E. coli* food, which may bias the range of genotypes obtained. We are thus in need of a new *C. elegans* isolation protocol, which allows us to obtain a larger number of individuals from a particular location for assessment of exhaustive sampling and reliable inference of population size. Moreover, the protocol needs to be fast and should not require an attractant to ensure unbiased sampling. Such an isolation protocol may be possible with the help of a series of sieves with different mesh sizes, as commonly used in studies of soil inhabitants. Without doubt, the current exploration of *C. elegans* ecology is still in its infancy and will clearly benefit from a more systematic and exhaustive sampling program in the future.

## Data Archiving

Genotype and phenotype data have been submitted to Dryad (doi:10.5061/dryad.9j850).

## Supporting Information

**Table S1**. Analysed *C. elegans* strains with information on corresponding subpopulation, genotype and repeat numbers at each microsatellite locus.

**Table S2.** Genotypes and corresponding repeat numbers on analysed loci found among the natural *C. elegans* isolates.

**Table S3.** Number and frequency of genotypes in each subpopulation and within the whole dataset of all analysed natural isolates (overall).

**Table S4.** Comparison of population growth rates of natural *C*. *elegans* isolates.

**Table S5.** Statistical analysis of population growth of natural *C*. *elegans* isolates in comparison to the wildtype N2.

**Table S6.** Comparison of choice behaviour of natural *C*. *elegans* strains.

**Table S7.** Choice behaviour of natural *C*. *elegans* isolates in comparison to the wild type strain N2.

**Table S8.** Correlations between population growth rate and choice behaviour in natural *C*. *elegans* isolates.
